# Keeping it cool: Soil sample cold pack storage and DNA shipment up to 1 month does not impact metabarcoding results

**DOI:** 10.1002/ece3.6219

**Published:** 2020-03-31

**Authors:** Camille S. Delavaux, James D. Bever, Erin M. Karppinen, Luke D. Bainard

**Affiliations:** ^1^ Department of Ecology and Evolutionary Biology The University of Kansas Lawrence KS USA; ^2^ Kansas Biological Survey The University of Kansas Lawrence KS USA; ^3^ Swift Current Research and Development Centre Agriculture and Agri‐Food Canada Swift Current SK Canada

**Keywords:** bacteria, fungi, metabarcoding, microbial biology

## Abstract

With the advances of sequencing tools, the fields of environmental microbiology and soil ecology have been transformed. Today, the unculturable majority of soil microbes can be sequenced. Although these tools give us tremendous power and open many doors to answer important questions, we must understand how sample processing may impact our results and interpretations. Here, we test the impacts of four soil storage methods on downstream amplicon metabarcoding and qPCR analyses for fungi and bacteria. We further investigate the impact of thaw time on extracted DNA to determine a safe length of time during which this can occur with minimal impact on study results. Overall, we find that storage using standard cold packs with subsequent storage at −20°C is little different than immediate storage in liquid nitrogen, suggesting that the historical and current method is adequate. We further find evidence that storage at room temperature or with aid of RNAlater can lead to changes in community composition and in the case of RNAlater, lower gene copies. We therefore advise against these storage methods for metabarcoding analyses. Finally, we show that over 1 month, DNA extract thaw time does not impact diversity or qPCR metrics. We hope that this work will help researchers working with soil bacteria and fungi make informed decisions about soil storage and transport to ensure repeatability and accuracy of results and interpretations.

## Graphical abstract

Here, we test the impacts of four soil storage methods on downstream amplicon metabarcoding and qPCR analyses for fungi and bacteria and the impact of thaw time on extracted DNA to determine a safe length of time during which this can occur with minimal impact on study results. Overall, we find that storage using standard cold packs with subsequent storage at −20°C is little different than immediate storage in liquid nitrogen, suggesting that the historical and current method is adequate. We also show that over 1 month, DNA extract thaw time does not impact diversity or qPCR metrics.

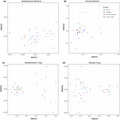

## 1 INTRODUCTION

In the last few decades, metagenomic tools have dramatically transformed the fields of environmental microbiology and soil ecology (Nesme et al., 2016). The advent of sequencing environmental DNA, the entire (sub) community in a field sample, represents an important advance in these fields. These tools have allowed researchers studying soil microbes directly from soil to analyze an increasing proportion of the previously unculturable microbiome. Although these tools have enabled an unprecedented view into soil microbial diversity, we must use caution when interpreting results. To wield these powerful tools responsibly, we must understand how robust environmental sequencing results are to methods of soil storage and processing. This will allow us to make decisions we know do not alter results, or at the very least, understand how they do so.

Two important sample processing choices that may alter experimental results are (a) the type of soil storage method and (b) DNA extract thaw time, the length of time for which extracted DNA is transported and left to thaw. To date, only a handful of studies have examined consequences of soil storage methods on study results (i.e., temperature, absolute ethanol, freeze‐drying, RNAlater, PLFA) on targets such as DNA, RNA, bacteria, fungi, and arbuscular mycorrhizal fungi (Brandt, Breidenbach, Brenzinger, & Conrad, 2014; Cui et al., 2014; Harry, Gambier, & Garnier‐Sillam, 2000; Klammer, Mondini, & Insam, 2005; Lauber, Zhou, Gordon, Knight, & Fierer, 2010; Rissanen, Kurhela, Aho, Oittinen, & Tiirola, 2010; Rubin et al., 2013; Tzeneva et al., 2009; Weißbecker, Buscot, & Wubet, 2017). These studies have broadly found little impact of storage method, but they do not thoroughly explore common storage practices used in the field and focus overwhelmingly on bacteria. This leaves researchers with an unclear understanding of how their choice of soil storage will impact study conclusions and interpretations. Most studies find no impact of soil sample storage over short periods of time under 1 month at temperatures from 4 to −80°C (Brandt et al., 2014; Harry et al., 2000; Klammer et al., 2005; Lauber et al., 2010; Schnecker, Wild, Fuchslueger, & Richter, 2012; Tatangelo, Franzetti, Gandolfi, Bestetti, & Ambrosini, 2014; Weißbecker et al., 2017). Some studies compare freeze‐drying in addition to storage at different temperatures (Cui et al., 2014; Klammer et al., 2005; Tatangelo et al., 2014; Weißbecker et al., 2017). Nonetheless, most of these studies fail to investigate several practical and commonly used storage methods besides different temperatures (Brandt et al., 2014; Lauber et al., 2010; Rubin et al., 2013; Tatangelo et al., 2014). For example, liquid nitrogen (N_2_), thought to be the most effective storage method and the relevant “control” comparison, is not analyzed in any of these papers. RNAlater, another potential method to store soil samples without refrigeration, has received only moderate attention, with mixed results (Rissanen et al., 2010; Schnecker et al., 2012). Rissanen et al. (2010) found that storage in RNAlater decreased nucleic acid yields drastically at all temperatures (−80°C to 4°C), while Schnecker et al. (2012) found no significant difference between RNAlater storage and other study treatments (−20°C, 4°C, direct extraction from fresh soil), although these authors were looking at total phospholipid fatty acid (PLFA) content and not nucleic acids. In addition, Nilsson et al. (2019) caution against using RNAlater for high‐throughput analyses, as it fails for complex substrates, although this is for use with RNA. Finally, an extremely low proportion of these studies investigate storage impacts on fungi (Cui et al., 2014; Schnecker et al., 2012; Weißbecker et al., 2017), with the majority focusing on bacteria (Brandt et al., 2014; Harry et al., 2000; Lauber et al., 2010; Rissanen et al., 2010; Rubin et al., 2013; Tatangelo et al., 2014; Tzeneva et al., 2009). The few that include fungi do not test the impact on common amplicon and qPCR metabarcoding analyses (Cui et al., 2014; Schnecker et al., 2012). Nonetheless, these kinds of analyses are still the predominant approach in most soil microbial studies.

In addition to soil storage method, DNA extract thaw time may be important in determining study outcomes due to DNA degradation. In our experience, isolated field locations often ship samples of extracted DNA for library preparation to equipped laboratories. There is often much less regulation surrounding the shipment of DNA as compared to live soil, making the option of sending extracted DNA much more practical. For example, the United States Department of Agriculture (USDA) currently has no regulations for extracted DNA. To our knowledge, the impact of DNA extract thaw time on samples (DNA degradation) has not been studied before. Nonetheless, this is an important issue that may have consequences for study results and interpretation. Researcher decisions involving shipment speed or the length of time extracted DNA will travel should be made based on an accurate understanding of the impact of alternatives on work and conclusions.

Here, we assess the impact of four different soil storage methods and extracted thaw time on soil sample DNA extracts for both bacterial and fungal communities. The storage methods range from the bare minimum (room temperature), to the assumed best method (liquid nitrogen), but also includes the most common method in the field (cooler with cold packs) as well as an additional potentially useful method in situations lacking facilities with fridges or freezers (RNAlater). In addition, we aim to get a better understanding of the impact of DNA extract thaw time to ultimately suggest a “safe time” in which this can occur: a time up until which thawing DNA extract will not degrade and impact results. We assess soil storage method and DNA extract thaw time in terms of commonly used community composition, diversity, and gene abundance metrics for microbial metabarcoding, specifically amplicon sequencing, including both OTU based analyses and qPCR gene copy measurements. We hope that this study will help researchers make more informed decisions about soil storage methods and transportation of extracted DNA, ultimately resulting in reliable and reproducible results.

## 2 MATERIALS AND METHODS

Here, we used amplicon sequencing to assess the consequences of soil storage method and thaw time on DNA extracts. We included viable and common options of sample storage in soil ecology, including room temperature, cold packs (referred to herein as “cooler”), liquid nitrogen, and RNAlater. We then examined these different soil storage methods to determine the impact this would have on subsequently extracted DNA composition (community composition and diversity metrics) and quantity (qPCR). We further investigated the impacts of DNA extract thaw time, testing the same community and quantity metrics. All amplicon metabarcoding analyses were conducted for bacteria and fungi, as these are two major microbial groups of interest in soil.

### 2.1 Sample collection

We collected soil from two remnant prairie locations in North America, with one in Kansas (KS), USA, and the other in Saskatchewan (SK), Canada in September 2018. Here, we defined remnant as having experienced minimal anthropogenic disturbance and therefore representing mostly intact native prairie ecosystems. We chose remnant prairie to represent soils with high plant and microbial diversity. The Kansas prairie, Welda, is in Anderson County Prairie Preserve, which is part of the University of Kansas Field Station sites. This site has a mean annual temperature of 13.14°C and a mean annual precipitation of 104.08 cm. The Saskatchewan prairie is part of the Swift Current Research and Development Centre research farm. This site has a mean annual temperature of 9.7°C and mean annual precipitation of 36.56 cm.

We collected a total of 12, 2 ml soil samples per site to be stored in cryogenic tubes, for a total of 24 samples. These samples were from a large homogenized soil sample from each site (total of 2 large samples). At each site, this homogenized soil sample was formed by sampling a central core and four cores at 90° (corners of a square) 3 m apart from each other around the central core. All cores were taken at a depth of 10 cm, which included soil horizons A and B. We stored soil using four methods, room temperature, cooler, liquid nitrogen, and RNAlater (Ambion), in replicates of three per location. For RNAlater samples, we added 6 ml of RNAlater to each tube. For the liquid nitrogen storage method, we used a vapor shipper (CBS transport SC4/2V series; Horsham, PA). After 24 hr in each respective soil storage method, we stored all samples in their permanent storage method, or post‐transport storage method in the laboratory, that most closely aligned with protocols used in practice (Table 1).

**TABLE 1:** Sampling and storage for each replicate sample, repeated at Anderson County Prairie Preserve in Kansas, USA and at Conway pasture in Swift Current, Saskatchewan, Canada

Storage method	Permanent storage method
Liquid nitrogen	−80
Liquid nitrogen	−80
Liquid nitrogen	−80
Cooler	−20
Cooler	−20
Cooler	−20
RNAlater	Room temp
RNAlater	Room temp
RNAlater	Room temp
Room temp	Room temp
Room temp	Room temp
Room temp	Room temp

### 2.2 Library preparation

We extracted DNA from 0.25 g of soil in triplicate for each sample using the DNeasy PowerSoil Pro Kit (Qiagen) 10 days after sample collection. DNA was extracted in batches of 12 samples using an automated system (QIAcube, Qiagen) for all samples in Swift Current, Saskatchewan, and in one batch of 36 samples using the standard (manual) protocol for all Kansas samples. Kansas sample DNA extracts were sent to Saskatchewan for all downstream amplicon and qPCR preparation and analysis in a vapor shipper (CBS transport SC4/2V series; Horsham, PA). Extracted DNA was quantified using a Qubit dsDNA BR Assay Kit (Thermo Fisher) and NanoDrop 1,000 spectrophotometer (Thermo Fisher). We conducted bacterial and fungal (a) amplicon sequencing and (b) qPCR measurements on all storage methods. For amplicon sequencing, DNA extracts were shipped on dry ice to the Genome Quebec Innovation Center for amplicon library preparation and Illumina MiSeq sequencing (see Appendix S1 for detailed methods) of the bacterial 16S rRNA gene using primers 515‐F and 806‐R (Caporaso et al. 2011) and fungal ITS1 region using primers ITS1F and 58A2R (Martin and Rygiewicz 2005). The raw amplicon sequencing dataset is available in the NCBI Sequence Read Archive under BioProject ID: PRJNA575860. Full descriptions of amplicon library preparation and Illumina MiSeq sequencing are available in Supplementary Methods 1 and 2 (Appendix S1).

For the qPCR assays, we used the same primers as used in amplicon sequencing to quantify the abundance of 16S (515‐F and 806‐R) and ITS1 (ITS1F and 58A2R) copies in the sample DNA extracts. The 25 µl reactions consisted of 5 µl of standardized DNA (5 ng/µl), 2 X Rotor‐gene SYBR Green PCR master mix (Qiagen), 0.2 µM of each primer, 0.5 µM BSA (Invitrogen), and nuclease‐free water. Reactions were run on a Rotor‐Gene Q real‐time PCR cycler (Qiagen) using the Rotor‐Disc 100 (Qiagen) format. The qPCR amplification conditions for bacteria (16S) consisted of an initial denaturing for 3 min at 95°C, then 40 cycles of 45 s denaturing at 95°C and 1 min annealing at 60°C, and extension at 72°C, followed by a melt curve analysis. The qPCR amplification conditions for fungi (ITS1) consisted of an initial denaturing for 3 min at 95°C, then 40 cycles of 15 s denaturing at 95°C and 30 s annealing/extension at 59°C, followed by a melt curve analysis. A standard curve prepared in triplicate, ranging in concentration from 10^1^ to 10^7^ gene copies µl^−1^, was used to quantify gene copies in each sample DNA extract. Sample DNA extracts were amplified in duplicate and each run included controls lacking template.

To investigate the impact of thaw time on extracted DNA, we used only cooler samples due to the high sample number this test involves. We chose this method because cooler transport is the most common and cost‐effective method used in soil ecology, especially at remote field sites. For the DNA extract thaw time test, we took cooler sample DNA extracts stored at −20°C for 10 days and conducted sequencing and qPCR analyses at five different time intervals of thawing at room temperature (21°C) over 2 months (60 days) at 0, 3, 15, 30, and 60 days. All DNA yield (Qubit and NanoDrop) and qPCR values are reported in SI Table S1. From this table, we conclude that all sample DNA extracts are within a reasonable range of 260/280, with optimal ratio at 1.8 indicating pure DNA; the 260/230 values are relatively low compared to the optimal value of 2.0, but should be interpreted as a secondary measure of purity, with these low values likely an indicator of some contaminants in the 230 nm range.

### 2.3 Bioinformatics

Raw paired reads were processed using the UPARSE pipeline and USEARCH v.9 (Edgar, 2013). Paired reads were merged using the fastq_mergepairs command with a maximum of five (i.e., default) mismatches in the alignment. Merged reads were quality filtered using the command fastq_filter that discarded all reads that were less than 200 bp and those with expected errors >1. Sequences were dereplicated and the command cluster_otus was used to perform operational taxonomic unit (OTU) clustering (based on 97% similarity) and chimera filtering. Taxonomic identity was assigned using the RDP classifier (Wang, Garrity, Tiedje, & Cole, 2007) and 16S rRNA training set (version 16) for bacteria/archaea and ITS UNITE database for fungi (Kõljalg et al., 2013). Before all analyses, we filtered out all unmatching domains (including only archaea and bacteria in bacteria analyses; only fungi in fungal analyses). Operational taxonomic unit tables for each analysis were filtered to include OTUs with a minimum of 5 sequences. Finally, OTU tables for each analysis were normalized to the lowest number of sequences in a sample within the subset of data being analyzed using rrarefy from the R package vegan (Oksanen et al., 2013). Rarefaction curves are provided for each set of analyses in Figure S1 and indicate sufficient sequencing.

### 2.4 Statistical analyses

We conducted analyses to understand the impact of (a) soil storage method and (b) DNA extract thaw time on the composition (sequencing) and quantity (qPCR) of bacteria and fungi from our soil samples. We first analyzed soil storage method and DNA extract thaw time impact on bacterial and fungal community composition with permutational analysis of variance (PERMANOVA) tests. As part of this set of analyses, we also analyzed soil storage method and DNA extract thaw time on bacterial and fungal diversity metrics (OTU number, Simpson diversity, and Evenness) through general linearized mixed effects models (GLMMs). We also reran these models for each bacterial and fungal phylum for which we had enough available data (at least 40 observations). Finally, we analyzed soil storage method and DNA extract thaw time impact on bacterial and fungal gene copy quantity (qPCR) using GLMMs.

In our PERMANOVAs, we used either storage or time to predict the community composition of bacteria or fungi, as well as location and either storage or time's interaction with location. In the storage tests, we included the strata argument to account for nonindependency of sampling as replicate of storage. The strata argument (Oksanen et al., 2013) constrains permutations to a group and is the only option to account for this nonindependency of sampling in this package. In the DNA extract thaw time tests, we included only replication in the strata argument. We ran PERMANOVAs on all the data, and then within location (Kansas or Saskatchewan). When storage significantly impacted community composition in either location, we ran pairwise comparisons comparing liquid nitrogen storage to each other storage to obtain a better understanding of which storage methods alter community compositions. All PERMANOVAs were implemented in *adonis2* in vegan (Oksanen et al., 2013) using the morisita dissimilarity matrix, as it is robust to differences in sample size (Morisita, 1959). In our GLMMs to analyze diversity metrics, we used either storage or time to predict each diversity metric. Our metrics, used as response variables, included OTU number, Simpson diversity, or community Evenness. Simpson diversity was calculated using the diversity function within vegan; Evenness was calculated manually following the vignette for vegan by dividing Shannon diversity by the log of species number (Oksanen, 2013). Each GLMM model included the interaction of location and storage or location and time, with the random effect of storage replicate nested within storage type nested with location. We repeated PERMANOVAs and GLMMs to analyze diversity metrics for each bacterial and fungal phylum for which we had enough available data. We were able to run these analyses for the bacterial phyla of Actinobacteria, Planctomycetes, and Proteobacteria and for the fungal phyla of Ascomycetes, Basidiomycetes, and Zygomycetes.

In our GLMMs to assess qPCR results, we used 16S copies per gram of dry soil for bacteria and ITS1 copies per gram of dry soil for fungi as the response variables. Each model included the interaction of location and storage or location and time, with the random effect of storage replicate nested within storage type nested with location. All GLMM models were run using the *lme4* package (Bates, Maechler, Bolker, & Walker, 2015), and all statistical analyses were carried out in R version 3.4.1 (R Core Team, 2019). We report only significant results in the Results Section, but all results can be found in Supplementary Tables (Tables S1–S5).

After processing our data, we noticed that the qPCR results showed a strong trend in the sample DNA extracts from Kansas. Gene copies resulting from qPCR were higher in samples that were extracted later in time. This is because in Kansas, the DNA extractions for 36 samples were conducted in one batch DNA extraction. To test this effect statistically, we reran each GLMM model (OTU richness, Simpson diversity, community Evenness, or qPCR gene copy quantity) as well as PERMANOVA model for the entire bacterial and fungal datasets using order as a covariate instead of time or storage; the random effect structures and use of the strata argument remained the same as in other analyses.

## 3 RESULTS

### 3.1 Storage

**FIGURE 1:**
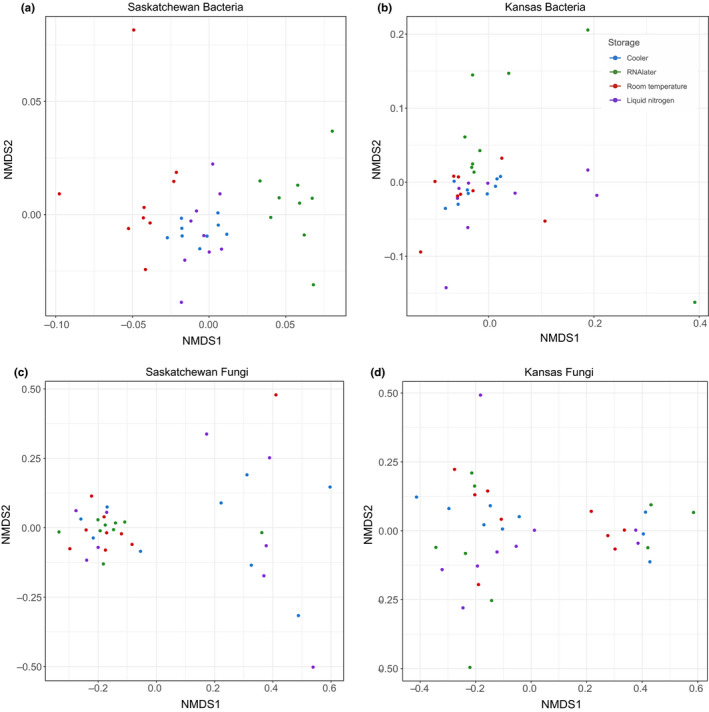
NMDS of bacterial (a, b) and fungal (c, d) communities coded by storage (color) for each location. These plots show that there is some differentiation of communities based on storage, particularly in Saskatchewan bacteria

For bacterial community composition, we found an impact of storage method in both locations (Table 2A, KS *p* = .04, SK *p* = 2.00E−04; Figure 1a,b), as well as a significant interaction between storage and location (Table 2A, *p* = .03). When comparing each storage method to liquid nitrogen, the assumed best method, we found a significant impact of storage in room temperature (Table 2A, *p* = 4.00E−03) and in RNAlater (Table 2A, *p* = 2.00E−04) in Saskatchewan samples; we found no impact of storage between liquid nitrogen and each other method in Kansas samples (Table 2A). Nonetheless, we found that in both Kansas and Saskatchewan, storage between cooler and liquid nitrogen does not impact community composition (Table 2A, KS *p* = .19, SK = .14). We found that fungal community composition was not affected by storage method in Saskatchewan samples, but was in KS samples (Table 2, All samples *p* = .46, KS *p* = 1.40E−03, SK *p* = .30; Figure 1c,d). Within Kansas samples, we found a significant impact of storage in room temperature (*p* = .04) and in RNAlater (Table 2A, *p* = .03) as compared to liquid nitrogen; again, we did not find a significant impact of storage on community composition when comparing cooler and liquid nitrogen storage (Table 2A, *p* = .44). When subsetting the data to specific bacterial and fungal phyla, community compositions across all samples were not impacted by storage, although we did find that within each location, storage is significant in certain phyla, particularly in bacterial phyla (Table S2a). When analyzing each phylum at each location with a significant storage effect comparing liquid nitrogen to each other storage method, we found that cooler is no different than liquid nitrogen, with the exception of Saskatchewan Planctomycetes (*p* = .01) and Basidiomycota (*p* = 4.00E−03).

**TABLE 2:** PERMANOVA results for fungal and bacterial community composition across a. storage methods, with analyses either including room temperature, cooler, liquid nitrogen, and RNAlater or each method compared to liquid nitrogen within location where storage across all storage methods was significant and b. degradation over time using cooler storage

	Parameter	*R* ^2^	*p* value
a. Storage Methods (all time 1)			
Bacteria all storage methods			
All samples	Storage:Loc	.00559	**.03**
KS	Storage	.23452	**.04**
SK	Storage	.75249	**2.00E−4**
Bacteria liquid nitrogen versus cooler			
KS	Storage	.14257	.19
SK	Storage	.12182	.14
Bacteria liquid nitrogen versus room temperature			
KS	Storage	.16891	.14
SK	Storage	.38047	**4.00E−03**
Bacteria liquid nitrogen versus RNA later			
KS	Storage	.0164	.54
SK	Storage	.74225	**2.00E−04**
Fungi all storage methods			
All samples	Storage:Loc	.03635	.46
KS	Storage	.15843	**1.4E−03**
SK	Storage	.10428	.30
Bacteria liquid nitrogen versus cooler			
KS		.05861	.44
Bacteria liquid nitrogen versus room temperature			
KS		.09516	**.04**
Bacteria liquid nitrogen versus RNA later			
KS		.12074	**.03**
b. Over Time (all cooler)			
Bacteria			
All samples	Time:Loc	−.00001	1
KS	Time	−.00108	.65
SK	Time	.06844	**.05**
Fungi			
All samples	Time:Loc	.01330	.29
KS	Time	.00532	1
SK	Time	.00487	.86

Each analysis is run for all samples and then for Kansas (KS) and Saskatchewan (SK) samples. Results significant at a level of *p* < .05 are in bold.

**FIGURE 2:**
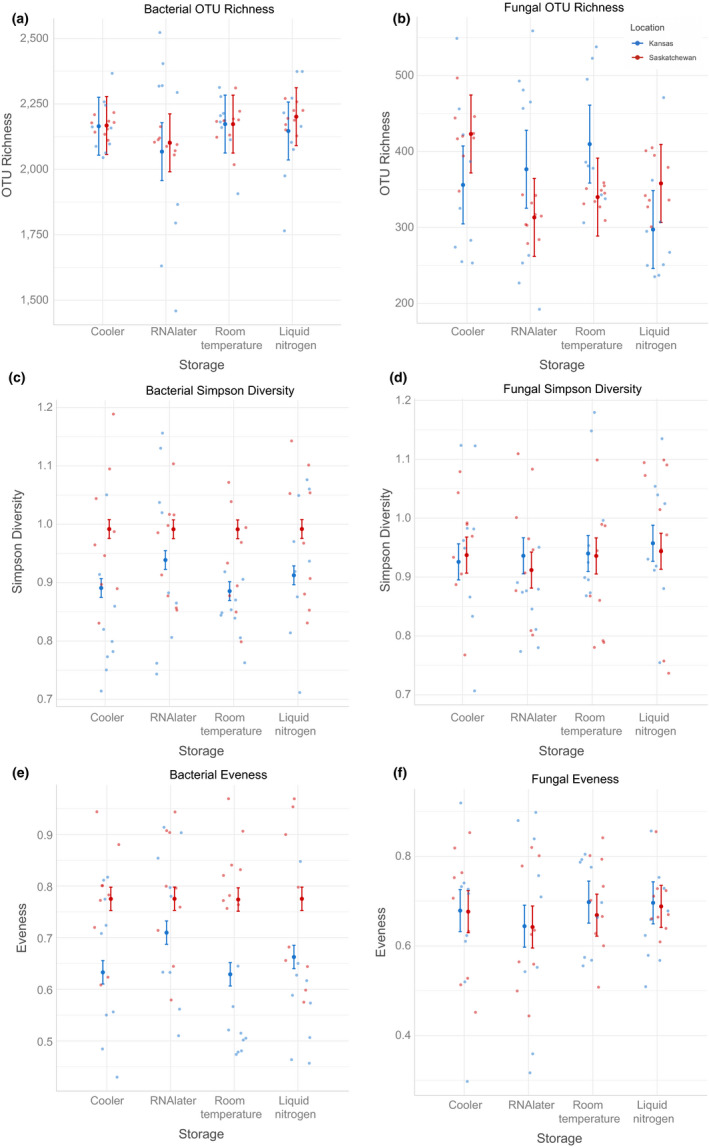
Diversity metrics of bacteria and fungi predicted by storage, with 95% confidence intervals. (a) bacterial OTU richness, (c) bacterial Simpson diversity, (e) bacterial Evenness, (b) fungal OTU richness, (d) fungal Simpson diversity, and (f) fungal Evenness. For bacteria, Simpson diversity (c) and Evenness (e) were greater in Saskatchewan than Kansas and greater for RNAlater than cooler. For fungi, OTU richness (b) is lower in Saskatchewan in RNAlater than room temperature as compared to Kansas

We found that diversity metrics (OTU richness, Simpson diversity and Evenness; Figure 2) were sensitive to storage, but responses varied mostly by location; diversity metrics were not impacted differentially by cooler compared to liquid nitrogen storage. For bacteria, Simpson diversity and Evenness were greater in Saskatchewan than Kansas (Table S3a; Simpson diversity *p* = 2.43E−09; Evenness *p* = 1.52E−09) and greater in RNAlater than liquid nitrogen storage (Table S3a; Simpson diversity *p* = .03; Evenness *p* = 4.37E−03; Figure 2c,e). We also saw an interaction between location and RNAlater, with RNAlater storage resulting in lower Evenness (Table S3a; *p* = .04) in Kansas, but not Saskatchewan samples (Figure 2c,e). For fungi, we only found significant results when looking at OTU number, with OTU number being greater in RNAlater (*p* = .02) and room temperature (*p* = 2.71E−03) overall and lower in Saskatchewan compared to Kansas in RNAlater (Table S3a; *p* = .02) and room temperature (Table S3a; *p* = .02; Figure 2b). All diversity metrics are depicted in Figure 2(a–e) and Table S3a.

For diversity results of specific phyla, we again found no evidence of differential effects of cooler storage versus liquid nitrogen. We found that bacteria are most impacted by other storage methods, while fungi are almost not (Table S4a). RNAlater resulted in both lower (Actinobacteria OTU richness, *p* = 2.85E−03; Planctomycetes OTU richness, *p* = .01) and higher (Proteobacteria Simpson diversity, *p* = 5.38E−06; Proteobacteria Evenness, *p* = 4.50E−05) diversity metrics as compared to cooler storage for bacteria. For fungi, RNAlater was only a significant predictor of Evenness within the Ascomycota (*p* = .03). Moreover, there was an RNAlater by location interaction in both bacteria and fungi, with RNAlater reducing most diversity metrics (Proteobacteria Simpson diversity, *p* = 4.98E−04; Ascomycota OTU richness, *p* = .03; Basidiomycota OTU number, *p* = 7.18E−03) compared to cooler storage in Saskatchewan, with the exception of Actinobacteria, which increased diversity metrics (OTU richness, *p* = .03). Room temperature significantly predicted an increase in the Simpson diversity of Proteobacteria (*p* = 3.37E−04). In addition, there was a room temperature by location interaction in two phyla of fungi, with both Ascomycota (OTU richness, *p* = .02) and Basidiomycota (OTU richness, *p* = .04) predicting a decrease in diversity metrics. Finally, we found that location was important in determining these results, much like in the broader analysis (see Table S4a for detailed results).

**FIGURE 3:**
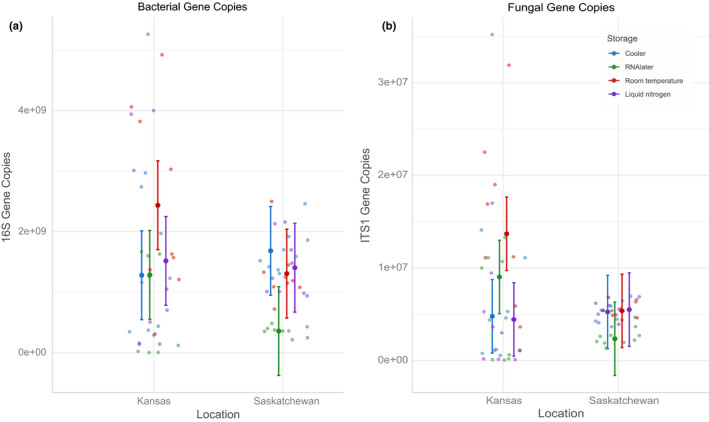
Gene copy number for 16S (bacteria; a) and ITS (fungi; b) based on storage and location, with 95% confidence intervals. Lower gene copies of both bacteria (a, *p* = 1.62E−08) and fungi (b, *p* = 2.55E−04) were found in Saskatchewan for samples stored in RNAlater

Storage in cold packs versus liquid nitrogen did not impact qPCR (quantity) results. However, storage in RNAlater reduced qPCR estimates of gene copy number for both fungi and bacteria in Saskatchewan (Table 3; bacterial *p* = 1.62E−08, fungal *p* = 2.66E−04; Figure 3a,b).

**TABLE 3:** qPCR GLM results

Parameter	Estimate (coefficient)	*SE*	*t*‐value	*p* value
a. Storage Methods (all time 1)				
Bacteria (16S)				
All samples				
Intercept	1.52E+09	3.67E+08	4.133	**3.58E−05**
Location (SK)	−1.12E+08	5.19E+08	−0.217	.83
Cooler	−2.37E+08	5.19E+08	−0.457	.65
RNAlater	−2.31E+08	5.19E+08	−0.445	.66
Room temperature	9.18E+08	5.19E+08	1.769	.08
Location * cooler	5.16E+08	7.34E+08	0.703	.48
Location * RNAlater	−8.15E+08	7.34E+08	−1.11	.27
Location * room temperature	−1.02E+09	7.34E+08	−1.384	.17
KS				
Intercept	1.52E+09	5.02E+08	3.02	**2.53E−03**
Cooler	−2.37E+08	7.10E+08	−0.334	.74
RNAlater	−2.31E+08	7.10E+08	−0.325	.74
Room temperature	9.18E+08	7.10E+08	1.293	.20
SK				
Intercept	1.41E+09	1.31E+08	10.724	**<2e−16**
Cooler	2.79E+08	1.85E+08	1.504	.13
RNAlater	−1.05E+09	1.85E+08	−5.648	**1.62E−08**
Room temperature	−9.80E+07	1.85E+08	−0.529	.60
Fungi (ITS)				
All samples				
Intercept	4.44E+06	1.99E+06	2.234	**.03**
Location (SK)	1.07E+06	2.81E+06	0.38	.70
Cooler	3.42E+05	2.81E+06	0.122	.90
RNAlater	4.59E+06	2.81E+06	1.635	.10
Room temperature	9.25E+06	2.81E+06	3.295	**9.85E−04**
Location * cooler	−6.02E+05	3.97E+06	−0.152	.88
Location * RNAlater	−7.74E+06	3.97E+06	−1.95	.05
Location * room temperature	−9.39E+06	3.97E+06	−2.365	**.02**
KS				
Intercept	4.44E+06	2.79E+06	1.589	.11
Cooler	3.42E+05	3.95E+06	0.087	.93
RNAlater	4.59E+06	3.95E+06	1.163	.25
Room temperature	9.25E+06	3.95E+06	2.344	**.02**
SK				
Intercept	5,502,222	361,006	15.241	**3.40E−07**
Cooler	−260000	510,539	−0.509	.62
RNAlater	−3153333	510,539	−6.176	**2.66E−04**
Room temperature	−141111	510,539	−0.276	.79
b. Over Time (all cooler)				
Bacteria (16S)				
All samples				
Intercept	1.20E+09	1.51E+08	7.926	**2.00E−15**
Location (SK)	4.27E+08	2.14E+08	2.001	**.05**
Time	−4.27E+06	4.91E+06	−0.869	.38
Location * time	−2.28E+06	6.94E+06	−0.328	.74
KS				
Intercept	1.20E+09	1.98E+08	6.045	**1.00E−09**
Time	−4.27E+06	6.43E+06	−0.663	.51
SK				
Intercept	1.62E+09	8.01E+07	20.286	**<2e−16**
Time	−6.54E+06	2.60E+06	−2.515	**.01**
Fungi (ITS)				
All samples				
Intercept	4.72E+06	7.50E+05	6.291	**4.00E−05**
Location (SK)	3.65E+05	1.06E+06	0.344	.70
Time	2.06E+04	2.25E+04	−0.917	.36
Location * time	7.10E+02	3.17E+04	−0.022	.98
KS				
Intercept	4.72E+06	1.04E+06	4.532	**4.00E−03**
Time	−2.06E+04	3.09E+04	−0.666	.51
SK				
Intercept	5.08E+06	2.19E+05	23.171	**<2e−16**
Time	−2.13E+04	7.13E+03	−2.986	**3.00E−03**

Note

GLM results for fungal and bacterial gene copies resulting from qPCR measurements across a. storage methods, including room temperature, cooler, liquid nitrogen, and RNAlater (with liquid nitrogen as the storage reference) and b. degradation over time using cooler storage. Each analysis is run for all samples and then for Kansas (KS) and Saskatchewan (SK) samples. Results significant at a level of *p* < .05 are in bold.

### 3.2 DNA extract thaw time

Overall, entire community composition was only impacted by DNA extract thaw time in Saskatchewan bacteria (Table 2B, *p* = .05). Within phyla, community composition was impacted by time in two bacterial phyla. Specifically, the community composition of Actinobacteria (All samples *p* = 3.40E−03) and Proteobacteria (All samples *p* = 2.00E−04) were significantly impacted by time (Table S2b). In contrast, diversity metrics including OTU number, Simpson diversity, and Evenness were not impacted by time overall or within phyla (Tables S3 and S5).

**FIGURE 4:**
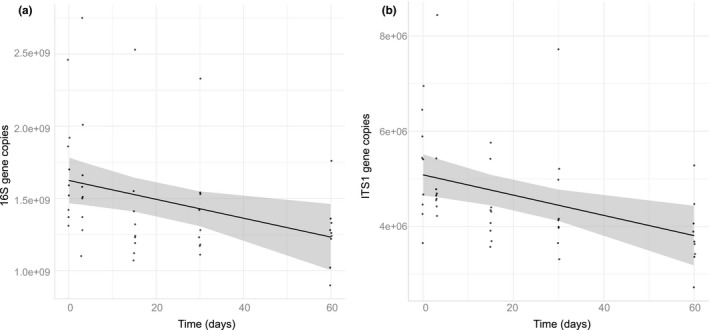
Gene copy quantity, as measured by qPCR, decreases with longer thaw time in both bacterial (a) and fungal (b) samples in Saskatchewan (bacteria *p* = .01; fungi, *p* = 3.00E–03). This is no longer true when removing the last time point of 60 days (bacteria *p* = .11; fungi, *p* = .13)

However, time impacted both bacterial and fungal gene copies (quantity) in Saskatchewan only. In both bacteria (*p* = .01) and fungi (*p* = 3.00E−03) in Saskatchewan, quantity decreased with time (Table 3B). To determine at what time point this decrease in quantity occurs, we reran the analyses progressively removing the oldest time point. After removing the oldest time point (60 days), we determined that this impact of time in Saskatchewan disappears (Figure 4, bacterial *p* = .11, fungal *p* = .13), suggesting that qPCR results are unimpacted by DNA extract thawing over 1 month or 30 days.

### 3.3 Order of DNA extraction batch

We found that community composition of fungi (Table S5a, *p* = 1.20E−03) was impacted by DNA extraction order in a batch for Kansas samples (36 samples in one batch). In contrast, none of our diversity metrics were significantly impacted by this order for fungi or bacteria (Table S5b). When looking at qPCR results, we found a positive correlation between qPCR gene copy quantity and order for bacteria (Table S5c, *p* = 5.87E−08) and fungi (Table S5c, *p* = 2.99E−05) for Kansas samples, with samples prepared later in the batch having a higher number of gene copies.

## 4 DISCUSSION

Here, we find broad support for the common use of cold packs in coolers for soil storage prior to DNA extraction, as there was little difference with immediate immersion in liquid nitrogen for downstream amplicon and qPCR analyses for fungi and bacteria. We further find that DNA extract thaw time can alter community composition and qPCR results, but found that 30 days of thaw time does not alter qPCR results or diversity metrics (bacteria, fungi, and phyla within). Overall, the finding of little difference between liquid nitrogen and the conventional cooler method is positive for soil ecology, as most studies transport soils in a cooler and do not have the capacity to store them in liquid nitrogen. Further, our finding that 30 days of DNA extract thaw time does not impact qPCR results or diversity metrics  is helpful for many studies that ship extracted DNA and may not be able to do so with temperature control.

Liquid nitrogen soil storage for transport to the laboratory is the standard method to preserve nucleic acids (Nilsson et al., 2019; Weißbecker et al., 2017). Therefore, we expected this method to yield the best results. Nonetheless, we show that in terms of the most commonly used analyses conducted with molecular data in the field of microbial ecology, namely community composition, diversity, and qPCR analyses, liquid nitrogen is little different than cooler storage. Storage does not impact overall community composition, in agreement with studies using both amplicon sequencing (Brandt et al., 2014; Lauber et al., 2010; Weißbecker et al., 2017) and community fingerprinting methods (Klammer et al., 2005; Tatangelo et al., 2014), but we find that storage may matter in finer grain analyses of phyla, in agreement with Rubin et al. (2013) and Rissanen et al. (2010). This is in contrast to some community fingerprinting studies that find storage does impact community composition (Cui et al., 2014; Tzeneva et al., 2009). Storage may impact diversity metrics, but understanding which storage method is better may be location dependent. Temperature, precipitation, and pH have been shown to impact microbial structure in both bacteria and fungi (Fierer, Strickland, Liptzin, Bradford, & Cleveland, 2009; Lauber, Strickland, Bradford, & Fierer, 2008; Newsham et al., 2016; Rincón et al., 2015; Zhou et al., 2016) and may alter storage effectiveness. Therefore, future work could incorporate soil chemical properties and environmental data to help understand location or soil physicochemical differences. Previous studies have also found storage method to impact diversity metrics in bacteria (Rubin et al., 2013; Weißbecker et al., 2017) and fungi (Cui et al., 2014). Finally, we see no difference of impact of storage on qPCR results between liquid nitrogen and cooler storage in our study which aligns with previous work (Brandt et al., 2014). This lack of a result is again positive for field ecology because there are no differences in broad fungal or bacterial results between liquid nitrogen and cooler storage; the traditional and much more cost‐effective method of cooler storage is likely sufficient for molecular studies for both bacteria and fungi.

Here, we find evidence that soil storage in RNAlater leads to sample degradation. When comparing samples stored in RNAlater to those stored in liquid nitrogen, we find several instances of community composition shifts. In addition, we find several differences with RNAlater in terms of diversity metrics, with conflicting directions (increasing or decreasing). We also show that RNAlater may lead to lower gene copy quantity from qPCR results, as seen here in the Saskatchewan results. This is indicative of degrading material and is not desirable. This is consistent with results from Rissanen et al. (2010) showing reduced yields with RNAlater and ethanol preservation. Tatangelo et al. (2014) also find reduced number of terminal restriction fragments in soil stored using LifeGuard (Qiagen), another solution‐based preservation method. Combining these inconsistent and negative results, we suggest future studies focused on DNA avoid using RNAlater.

Finally, to answer our second question looking at DNA extract thaw time, we can conclude that keeping extracted DNA at room temperature (e.g., for shipping purposes) is likely acceptable for up to 1 month. Nonetheless, leaving extracted DNA at room temperature can impact community composition and gene copy quantity. The impact on gene copy quantity only occurred in the Saskatchewan samples, possibly due to a difference in microbial community composition between sites. Time is not important in the Saskatchewan samples in predicting gene copy quantity when we remove the last time point (60 days), which suggests that keeping DNA at room temperature for up to 30 days has no impact on gene copy quantity results. We also find that commonly used diversity metrics are unaffected by DNA extract thaw time. Overall, we find that extracted DNA is not impacted by a month of room temperature storage in terms of gene copy quantity (qPCR) or diversity metrics; this result is positive for those scientists who work in remote locations and may worry about their sample DNA extracts thawing and degrading during travel.

Our incidental finding related to DNA extraction order in a batch may be useful to researchers extracting DNA broadly. We find that when working with large numbers of samples (here, 36), extraction in one batch is not advisable for consistency across samples. We find a strong relationship between order number in the batch and community composition as well as with gene copies. For gene copies, we find that later samples—those left in solutions longer—are those with greater gene copies, or yield. This suggests that at least for qPCR studies, a longer incubation time in solutions using the PowerSoil Pro kit may be optimal. However, we did not explicitly test this, and our results are from one site only (Kansas), so we cannot report an optimal time past recommended time for which to leave samples in kit solution.

Understanding sample processing is important for soil microbial molecular studies. Several steps may impact results and must be evaluated to make informed decisions. Here, we investigated impacts of soil sample storage method as well as DNA extract thaw time (DNA degradation). We urge further research into each step of soil sampling and processing to get a more complete understanding of how these decisions impact study results and interpretations. For example, several papers look at the impact of different bioinformatical pipelines on results (Bokulich et al., 2013; Cline, Song, Al‐Ghalith, Knights, & Kennedy, 2017; Egan et al., 2018). We are hopeful that this work will help scientists evaluate the costs and benefits of experimental approaches when studying soil bacteria and fungi. This work shows that in terms of community composition, diversity metrics and gene copy quantity, liquid nitrogen shows no clear difference from traditional cooler storage. In addition, researchers working abroad or in remote areas can be confident that for at least 1 month, thawing extracted DNA will not impact qPCR or commonly used diversity metric results.

## CONFLICT OF INTEREST

None declared.

## AUTHOR CONTRIBUTIONS

CSD, LDB, EMK, and JDB designing and performing the research; CSD, LDB, and JDB analyzing the data; and CSD, LDB, EMK, and JDB manuscript writing.

## Supporting information

Supplementary Material

Table S1

## Data Availability

The raw amplicon sequencing dataset is available in the NCBI Sequence Read Archive under BioProject ID: PRJNA575860.
