# Supplementary material for: Keeping it cool: Soil sample cold pack storage and DNA shipment up to 1 month does not impact metabarcoding results
Source: Ecol Evol. 2020 Mar 31;10(11):4652–64. doi: 10.1002/ece3.6219 (PMC7297747; doi:10.1002/ece3.6219)
Supplement: Supplementary file 1 — Supplementary Material [file ECE3-10-4652-s001.docx]

**Supplementary Information**

**Table S1.** DNA yield (Quibit; nanodrop) and qPCR results for each sample, alongside sample structure.

**Table S2.** PERMANOVA results for fungal and bacterial community composition for each phylum across a. storage methods, including room temperature, cooler, liquid nitrogen, and RNAlater, and b. degradation over time using cooler storage. Each analysis is run for all samples and then for Kansas (KS) and Saskatchewan (SK) samples. Results significant at a level of p < 0.05 are in bold.

| **a. Storage Methods (all time 1)** | | | |
| --- | --- | --- | --- |
|  | Parameter | R^2^ | *p* value |
| Bacteria (Actinobacteria) | | | |
| All Samples | storage:Loc | -0.00010 | 1 |
| KS | storage | 0.26775 | **9.8E-06** |
| SK | storage | 0.21848 | **0.05** |
| Bacteria (Planctomycetes) | | | |
| All Samples | storage:Loc | -0.00095 | 1 |
| KS | storage | 0.05587 | 0.71 |
| SK | storage | 0.33733 | **2.00E-04** |
| Bacteria (Proteobacteria) | | | |
| All Samples | storage:Loc | -0.00825 | 1 |
| KS | storage | 0.63049 | **2.00E-04** |
| SK | storage | 0.53557 | **2.00E-04** |
|  | | | |
| Fungi (Ascomycota) | | | |
| All Samples | storage:Loc | 0.02691 | 0.19 |
| KS | storage | 0.11742 | **0.01** |
| SK | storage | 0.06194 | 0.67 |
| Fungi (Basidiomycota) | | | |
| All Samples | storage:Loc | 0.03788 | 0.08 |
| KS | storage | 0.13404 | **0.04** |
| SK | storage | 0.18744 | **2.20E-03** |
| Fungi (Zygomycota) | | | |
| All Samples | storage:Loc | 0.02356 | 0.57 |
| KS | storage | 0.11111 | 0.91 |
| SK | storage | 0.02604 | 0.72 |
|  | | | |
| **b. Over Time (all cooler)** | | | |
| Bacteria (Actinobacteria) | | | |
| All Samples | time:Loc | 0.00013 | **3.40E-03** |
| KS | time | 0.04207 | 0.17 |
| SK | time | 0.00711 | 0.50 |
| Bacteria (Planctomycetes) | | | |
| All Samples | time:Loc | 0.00008 | 0.16 |
| KS | time | 0.01921 | 0.49 |
| SK | time | 0.05845 | 0.12 |
| Bacteria (Proteobacteria) | | | |
| All Samples | time:Loc | 0.00041 | **2.00E-04** |
| KS | time | 0.00711 | 050 |
| SK | time | 0.05407 | 0.22 |
|  | | | |
| Fungi (Ascomycota) | | | |
| All Samples | time:Loc | 0.00173 | 0.96 |
| KS | time | 0.00852 | 0.97 |
| SK | time | 0.00855 | 0.67 |
| Fungi (Basidiomycota) | | | |
| All Samples | time:Loc | 0.00000 | 1 |
| KS | time | 0.00009 | 1 |
| SK | time | 0.00115 | 1 |
| Fungi (Zygomycota) | | | |
| All Samples | time:Loc | -0.00062 | 0.89 |
| KS | time | -0.08827 | 0.83 |
| SK | time | -0.01755 | 0.93 |

**Table S3**. GLM results for fungal and bacterial diversity metrics (OTU numbers, Simpson diversity, and community Evenness) across a. storage methods, including room temperature, cooler, liquid nitrogen, and RNAlater (with liquid nitrogen as the storage reference) and b. degradation over time using cooler storage. Each analysis is run for all samples and then for Kansas (KS) and Saskatchewan (SK) samples. Results significant at a level of p < 0.05 are in bold.

| **a. Storage Methods (all time 1)** | | | | |
| --- | --- | --- | --- | --- |
| parameter | Estimate (coefficient) | Std. Error | t-value | *p* value |
| Bacteria (OTU number) | | | | |
| Intercept | 2139.78 | 55.98 | 38.222 | **<2e-16** |
| Location (SK) | 64.78 | 79.17 | 0.818 | 0.42 |
| cooler | 20.44 | 79.17 | 0.258 | 0.80 |
| RNAlater | -82.89 | 79.17 | -1.047 | 0.30 |
| room temperature | 41.56 | 79.17 | 0.525 | 0.60 |
| Location*cooler | -57.67 | 111.96 | -0.515 | 0.61 |
| Location*RNAlater | -21 | 111.96 | -0.188 | 0.85 |
| Location*room temperature | -76.56 | 111.96 | -0.684 | 0.50 |
| Bacteria (Simpson Diversity) | | | | |
| Intercept | 0.912431 | 0.008104 | 112.592 | **< 2e-16** |
| Location (SK) | 0.079466 | 0.011461 | 6.934 | **2.43E-09** |
| cooler | -0.021596 | 0.011461 | -1.884 | 0.06 |
| RNAlater | 0.026263 | 0.011461 | 2.292 | **0.03** |
| room temperature | -0.026866 | 0.011461 | -2.344 | **0.02** |
| Location*cooler | 0.021529 | 0.016208 | 1.328 | 0.19 |
| Location*RNAlater | -0.026668 | 0.016208 | -1.645 | 0.10 |
| Location*room temperature | 0.026359 | 0.016208 | 1.626 | 0.11 |
| Bacteria (Evenness) | | | | |
| Intercept | 0.66267 | 0.01133 | 58.503 | **< 2e-16** |
| Location (SK) | 0.11293 | 0.01602 | 7.05 | **1.52E-09** |
| cooler | -0.02867 | 0.01602 | -1.79 | 0.08 |
| RNAlater | 0.04733 | 0.01602 | 2.955 | **4.37E-03** |
| room temperature | -0.03409 | 0.01602 | -2.128 | **0.04** |
| Location*cooler | 0.02822 | 0.02265 | 1.246 | 0.22 |
| Location*RNAlater | -0.04791 | 0.02265 | -2.115 | **0.04** |
| Location*room temperature | 0.03265 | 0.02265 | 1.441 | 0.15 |
|  | | | | |
| Fungi (OTU Number) | | | | |
| Intercept | 296.556 | 25.76 | 11.512 | **< 2e-16** |
| Location (SK) | 58.556 | 36.431 | 1.607 | 0.11 |
| cooler | 61.444 | 36.431 | 1.687 | 0.10 |
| RNAlater | 83.667 | 36.431 | 2.297 | **0.02** |
| room temperature | 113.667 | 36.431 | 3.12 | **2.71E-03** |
| Location*cooler | 8.556 | 51.521 | 0.166 | 0.87 |
| Location*RNAlater | -124.222 | 51.521 | -2.411 | **0.02** |
| Location*room temperature | -127.556 | 51.521 | -2.476 | **0.02** |
| Fungi (Simpson Diversity) | | | | |
| Intercept | 0.957456 | 0.015302 | 62.569 | **< 2e-16** |
| Location (SK) | -0.013514 | 0.021641 | -0.624 | 0.54 |
| cooler | -0.031537 | 0.021641 | -1.457 | 0.15 |
| RNAlater | -0.0213 | 0.021641 | -0.984 | 0.33 |
| room temperature | -0.01776 | 0.021641 | -0.821 | 0.42 |
| Location*cooler | 0.024624 | 0.030605 | 0.805 | 0.42 |
| Location*RNAlater | -0.010879 | 0.030605 | -0.355 | 0.72 |
| Location*room temperature | 0.009706 | 0.030605 | 0.317 | 0.75 |
| Fungi (Evenness) | | | | |
| Intercept | 0.696316 | 0.023432 | 29.717 | **< 2e-16** |
| Location (SK) | -0.009109 | 0.033137 | -0.275 | 0.78 |
| cooler | -0.017604 | 0.033137 | -0.531 | 0.60 |
| RNAlater | -0.052489 | 0.033137 | -1.584 | 0.12 |
| room temperature | 0.001907 | 0.033137 | 0.058 | 0.95 |
| Location*cooler | 0.006722 | 0.046863 | 0.143 | 0.89 |
| Location*RNAlater | 0.007601 | 0.046863 | 0.162 | 0.87 |
| Location*room temperature | -0.018998 | 0.046863 | -0.405 | 0.69 |
|  | | | | |
| **b. Over Time (all cooler)** | | | | |
| Bacteria (OTU Number) | | | | |
| Intercept | 1945.3632 | 21.1731 | 91.879 | **3.00E-11** |
| Location (SK) | 8.5199 | 29.9432 | 0.285 | 0.79 |
| time | -0.6176 | 0.4544 | -1.359 | 0.18 |
| Location*time | 0.3082 | 0.6427 | 0.48 | 0.63 |
| Bacteria (Simpson Diversity) | | | | |
| Intercept | 8.97E-01 | 6.43E-03 | 139.42 | **5.00E-11** |
| Location (SK) | 9.54E-02 | 9.10E-03 | 10.488 | **7.00E-05** |
| time | 2.52E-05 | 1.16E-04 | 0.217 | 0.83 |
| Location*time | -3.12E-05 | 1.64E-04 | -0.189 | 0.85 |
| Bacteria (Evenness) | | | | |
| Intercept | 6.48E-01 | 7.51E-03 | 86.192 | **2.00E-10** |
| Location (SK) | 1.38E-01 | 1.06E-02 | 13.022 | **1.00E-05** |
| time | 2.82E-05 | 1.48E-04 | 0.19 | 0.85 |
| Location*time | -8.34E-05 | 2.10E-04 | -0.398 | 0.69 |
|  | | | | |
| Fungi (OTU Number) | | | | |
| Intercept | 344.21273 | 19.41618 | 17.728 | **< 2e-16** |
| Location (SK) | 24.50325 | 27.45862 | 0.892 | 0.38 |
| time | -0.53351 | 0.63101 | -0.845 | 0.40 |
| Location*time | 0.07238 | 0.89238 | 0.081 | 0.94 |
| Fungi (Simpson Diversity) | | | | |
| Intercept | 9.19E-01 | 2.06E-02 | 44.577 | **3.00E-10** |
| Location (SK) | 2.75E-02 | 2.92E-02 | 0.942 | 0.38 |
| time | -2.29E-04 | 4.90E-04 | -0.466 | 0.64 |
| Location*time | 4.03E-05 | 6.94E-04 | 0.058 | 0.95 |
| Fungi (Evenness) | | | | |
| Intercept | 0.6662125 | 0.020545 | 32.427 | **< 2e-16** |
| Location (SK) | 0.0257326 | 0.029055 | 0.886 | 0.38 |
| time | -0.0004139 | 0.0006677 | -0.62 | 0.54 |
| Location*time | 0.0001309 | 0.0009443 | 0.139 | 0.89 |

**Table S4.** GLM results for fungal and bacterial diversity metrics (OTU numbers, Simpson diversity, and community Evenness) for each phylum across a. storage methods, including room temperature, cooler, liquid nitrogen, and RNAlater (with liquid nitrogen as the storage reference) and b. degradation over time using cooler storage. Each analysis is run for all samples and then for Kansas (KS) and Saskatchewan (SK) samples. Results significant at a level of p < 0.05 are in bold.

| **a. Storage Methods (all time 1)** | | | | |
| --- | --- | --- | --- | --- |
| parameter | Estimate  (coefficient) | Std. Error | t-value | *p* value |
| *Bacteria (Actinobacteria)* | | | | |
| Bacteria (OTU number) | | | | |
| Intercept | 321.889 | 4.682 | 68.754 | **< 2e-16** |
| Location (SK) | -3.778 | 6.621 | -0.571 | 0.57 |
| cooler | 5.444 | 6.621 | 0.822 | 0.41 |
| RNAlater | -20.556 | 6.621 | -3.105 | **2.84E-03** |
| room temperature | 5.556 | 6.621 | 0.839 | 0.40 |
| Location*cooler | -2.556 | 9.363 | -0.273 | 0.79 |
| Location*RNAlater | 20.667 | 9.363 | 2.207 | **0.03** |
| Location*room  temperature | -1.444 | 9.363 | -0.154 | 0.88 |
| Bacteria (Simpson Diversity) | | | | |
| Intercept | 9.87E-01 | 4.63E-04 | 2130.429 | **< 2e-16** |
| Location (SK) | -1.30E-02 | 6.55E-04 | -19.827 | **< 2e-16** |
| cooler | 4.52E-04 | 6.55E-04 | 0.69 | 0.49 |
| RNAlater | -8.02E-05 | 6.55E-04 | -0.122 | 0.90 |
| room temperature | 8.00E-04 | 6.55E-04 | 1.222 | 0.23 |
| Location*cooler | 5.51E-06 | 9.27E-04 | 0.006 | 1.00 |
| Location*RNAlater | 5.03E-04 | 9.27E-04 | 0.543 | 0.59 |
| Location*room  temperature | 2.71E-04 | 9.27E-04 | 0.293 | 0.77 |
| Bacteria (Evenness) | | | | |
| Intercept | 0.8448468 | 0.0024267 | 348.144 | **< 2e-16** |
| Location (SK) | -0.0576602 | 0.0034319 | -16.801 | **1.38E-11** |
| cooler | 0.0019698 | 0.0034319 | 0.574 | 0.57 |
| RNAlater | 0.0041636 | 0.0034319 | 1.213 | 0.24 |
| room temperature | 0.0053274 | 0.0034319 | 1.552 | 0.14 |
| Location*cooler | -0.0041749 | 0.0048534 | -0.86 | 0.40 |
| Location*RNAlater | -0.0034507 | 0.0048534 | -0.711 | 0.49 |
| Location*room  temperature | -0.0007612 | 0.0048534 | -0.157 | 0.88 |
| *Bacteria (Planctomycetes)* | | | | |
| Bacteria (OTU number) | | | | |
| Intercept | 347.556 | 10.374 | 33.503 | **< 2e-16** |
| Location (SK) | 9.111 | 14.671 | 0.621 | 0.54 |
| cooler | 9.556 | 14.671 | 0.651 | 0.52 |
| RNAlater | -38 | 14.671 | -2.59 | **0.01** |
| room temperature | 11.111 | 14.671 | 0.757 | 0.45 |
| Location*cooler | -6.111 | 20.748 | -0.295 | 0.77 |
| Location*RNAlater | 23.667 | 20.748 | 1.141 | 0.26 |
| Location*room  temperature | -12.333 | 20.748 | -0.594 | 0.55 |
| Bacteria (Simpson Diversity) | | | | |
| Intercept | 0.9801027 | 0.0014166 | 691.852 | **< 2e-16** |
| Location (SK) | 0.0085689 | 0.0020034 | 4.277 | **6.44E-05** |
| cooler | 0.0013011 | 0.0020034 | 0.649 | 0.52 |
| RNAlater | -0.0011364 | 0.0020034 | -0.567 | 0.57 |
| room temperature | 0.001626 | 0.0020034 | 0.812 | 0.42 |
| Location*cooler | -0.0020187 | 0.0028333 | -0.712 | 0.48 |
| Location*RNAlater | 0.0006248 | 0.0028333 | 0.221 | 0.83 |
| Location*room  temperature | -0.0015185 | 0.0028333 | -0.536 | 0.59 |
| Bacteria (Evenness) | | | | |
| Intercept | 0.858927 | 0.005294 | 162.234 | **< 2e-16** |
| Location (SK) | 0.02591 | 0.007487 | 3.46 | **9.65E-04** |
| cooler | 0.004254 | 0.007487 | 0.568 | 0.57 |
| RNAlater | 0.003829 | 0.007487 | 0.511 | 0.61 |
| room temperature | 0.004428 | 0.007487 | 0.591 | 0.56 |
| Location*cooler | -0.007858 | 0.010589 | -0.742 | 0.46 |
| Location*RNAlater | -0.00352 | 0.010589 | -0.332 | 0.74 |
| Location*room  temperature | -0.003955 | 0.010589 | -0.374 | 0.71 |
| *Bacteria (Proteobacteria)* | | | | |
| Bacteria (OTU number) | | | | |
| Intercept | 258.111 | 7.081 | 36.453 | **< 2e-16** |
| Location (SK) | 34.889 | 10.014 | 3.484 | **3.06E-03** |
| cooler | 15.222 | 10.014 | 1.52 | 0.15 |
| RNAlater | 4.667 | 10.014 | 0.466 | 0.65 |
| room temperature | 16.222 | 10.014 | 1.62 | 0.12 |
| Location*cooler | -24.556 | 14.161 | -1.734 | 0.10 |
| Location*RNAlater | -5.778 | 14.161 | -0.408 | 0.69 |
| Location*room  temperature | -19.111 | 14.161 | -1.35 | 0.20 |
| Bacteria (Simpson Diversity) | | | | |
| Intercept | 0.859711 | 0.00461 | 186.478 | **< 2e-16** |
| Location (SK) | 0.103852 | 0.00652 | 15.928 | **3.09E-11** |
| cooler | 0.011806 | 0.00652 | 1.811 | 0.09 |
| RNAlater | 0.043487 | 0.00652 | 6.67 | **5.38E-06** |
| room temperature | 0.029577 | 0.00652 | 4.536 | **3.37E-04** |
| Location*cooler | -0.014571 | 0.009221 | -1.58 | 0.13 |
| Location*RNAlater | -0.040098 | 0.009221 | -4.349 | **4.98E-04** |
| Location*room  temperature | -0.024287 | 0.009221 | -2.634 | **0.02** |
| Bacteria (Evenness) | | | | |
| Intercept | 0.657488 | 0.005897 | 111.493 | **< 2e-16** |
| Location (SK) | 0.127708 | 0.00834 | 15.313 | **5.60E-11** |
| cooler | 0.013735 | 0.00834 | 1.647 | 0.12 |
| RNAlater | 0.046186 | 0.00834 | 5.538 | **4.50E-05** |
| room temperature | 0.028313 | 0.00834 | 3.395 | **3.70E-03** |
| Location*cooler | -0.017899 | 0.011794 | -1.518 | 0.15 |
| Location*RNAlater | -0.033329 | 0.011794 | -2.826 | **0.01** |
| Location*room  temperature | -0.014681 | 0.011794 | -1.245 | 0.23 |
|  | | | | |
| *Fungi (Ascomycota)* | | | | |
| Fungi (OTU Number) | | | | |
| Intercept | 80.444 | 13.396 | 6.005 | **9.93E-08** |
| Location (SK) | 44.444 | 18.945 | 2.346 | **0.02** |
| cooler | 32.778 | 18.945 | 1.73 | 0.09 |
| RNAlater | 40.889 | 18.945 | 2.158 | **0.03** |
| room temperature | 64 | 18.945 | 3.378 | **1.25E-03** |
| Location*cooler | -3.444 | 26.792 | -0.129 | 0.90 |
| Location*RNAlater | -58.556 | 26.792 | -2.186 | **0.03** |
| Location*room  temperature | -66.667 | 26.792 | -2.488 | **0.02** |
| Fungi (Simpson Diversity) | | | | |
| Intercept | 0.942211 | 0.012065 | 78.095 | **< 2e-16** |
| Location (SK) | -0.04073 | 0.017062 | -2.387 | **0.02** |
| cooler | 0.015279 | 0.017062 | 0.895 | 0.37 |
| RNAlater | -0.015182 | 0.017062 | -0.89 | 0.38 |
| room temperature | 0.009398 | 0.017062 | 0.551 | 0.58 |
| Location*cooler | -0.024972 | 0.02413 | -1.035 | 0.30 |
| Location*RNAlater | -0.016565 | 0.02413 | -0.686 | 0.49 |
| Location*room  temperature | -0.010101 | 0.02413 | -0.419 | 0.68 |
| Fungi (Evenness) | | | | |
| Intercept | 0.801765 | 0.01899 | 42.22 | **< 2e-16** |
| Location (SK) | -0.101903 | 0.026856 | -3.794 | **3.31E-04** |
| cooler | 0.003514 | 0.026856 | 0.131 | 0.90 |
| RNAlater | -0.062924 | 0.026856 | -2.343 | **0.02** |
| room temperature | -0.021809 | 0.026856 | -0.812 | 0.42 |
| Location*cooler | -0.035289 | 0.03798 | -0.929 | 0.36 |
| Location*RNAlater | 0.034682 | 0.03798 | 0.913 | 0.36 |
| Location*room  temperature | 0.017276 | 0.03798 | 0.455 | 0.65 |
| *Fungi (Basidiomycota)* | | | | |
| Fungi (OTU Number) | | | | |
| Intercept | 15 | 2.0581 | 7.288 | **6.80E-10** |
| Location (SK) | 8.4444 | 2.9105 | 2.901 | **5.14E-03** |
| cooler | 4.5556 | 2.9105 | 1.565 | 0.12 |
| RNAlater | 4.5556 | 2.9105 | 1.565 | 0.12 |
| room temperature | 2.8889 | 2.9105 | 0.993 | 0.32 |
| Location*cooler | 0.6667 | 4.1161 | 0.162 | 0.87 |
| Location*RNAlater | -11.4444 | 4.1161 | -2.78 | **7.18E-03** |
| Location*room  temperature | -8.7619 | 4.2606 | -2.056 | **0.04** |
| Fungi (Simpson Diversity) | | | | |
| Intercept | 0.72849 | 0.04339 | 16.789 | **< 2e-16** |
| Location (SK) | 0.15258 | 0.06136 | 2.487 | **0.02** |
| cooler | -0.0174 | 0.06136 | -0.284 | 0.78 |
| RNAlater | -0.08716 | 0.06136 | -1.42 | 0.16 |
| room temperature | -0.07553 | 0.06136 | -1.231 | 0.22 |
| Location*cooler | 0.03373 | 0.08678 | 0.389 | 0.70 |
| Location*RNAlater | 0.02669 | 0.08678 | 0.308 | 0.76 |
| Location*room  temperature | 0.02696 | 0.08983 | 0.3 | 0.77 |
| Fungi (Evenness) | | | | |
| Intercept | 0.68217 | 0.0399 | 17.097 | **< 2e-16** |
| Location (SK) | 0.10028 | 0.05643 | 1.777 | 0.08 |
| cooler | -0.0645 | 0.05643 | -1.143 | 0.26 |
| RNAlater | -0.16345 | 0.05643 | -2.897 | **5.21E-03** |
| room temperature | -0.08671 | 0.05643 | -1.537 | 0.13 |
| Location*cooler | 0.07447 | 0.0798 | 0.933 | 0.35 |
| Location*RNAlater | 0.14065 | 0.0798 | 1.763 | 0.08 |
| Location*room  temperature | 0.0307 | 0.0826 | 0.372 | 0.71 |
| *Fungi (Zygomycota)* | | | | |
| Fungi (OTU Number) | | | | |
| Intercept | 6.5 | 1.1314 | 5.745 | **1.67E-06** |
| Location (SK) | 1.5 | 1.2508 | 1.199 | 0.24 |
| cooler | 0.5 | 1.9596 | 0.255 | 0.80 |
| RNAlater | -1.5 | 1.9596 | -0.765 | 0.45 |
| room temperature | 3 | 1.3856 | 2.165 | **0.04** |
| Location*cooler | 0.8333 | 2.0997 | 0.397 | 0.69 |
| Location*RNAlater | 2.125 | 2.1082 | 1.008 | 0.32 |
| Location*room  temperature | -2.4444 | 1.5776 | -1.549 | 0.13 |
| Fungi (Simpson Diversity) | | | | |
| Intercept | 0.49699 | 0.042 | 11.833 | **1.38E-11** |
| Location (SK) | 0.19001 | 0.0486 | 3.909 | **1.10E-03** |
| cooler | 0.0457 | 0.07274 | 0.628 | 0.54 |
| RNAlater | 0.05014 | 0.07274 | 0.689 | 0.50 |
| room temperature | 0.0422 | 0.05243 | 0.805 | 0.43 |
| Location*cooler | -0.04167 | 0.08055 | -0.517 | 0.61 |
| Location*RNAlater | -0.07956 | 0.08081 | -0.985 | 0.34 |
| Location*room  temperature | -0.04537 | 0.06281 | -0.722 | 0.48 |
| Fungi (Evenness) | | | | |
| Intercept | 0.480768 | 0.047658 | 10.088 | **6.74E-12** |
| Location (SK) | 0.162865 | 0.052688 | 3.091 | **3.90E-03** |
| cooler | -0.006711 | 0.082547 | -0.081 | 0.94 |
| RNAlater | 0.013168 | 0.082547 | 0.16 | 0.87 |
| room temperature | -0.007067 | 0.058369 | -0.121 | 0.90 |
| Location*cooler | -0.006695 | 0.08845 | -0.076 | 0.94 |
| Location*RNAlater | -0.042348 | 0.088806 | -0.477 | 0.64 |
| Location*room  temperature | 0.004228 | 0.066456 | 0.064 | 0.95 |
|  | | | | |
| **b. Over Time (all cooler)** | | | | |
| *Bacteria (Actinobacteria)* | | | | |
| Bacteria (OTU Number) | | | | |
| Intercept | 326.91821 | 3.04892 | 107.224 | **3.00E-13** |
| Location (SK) | -7.91121 | 4.31182 | -1.835 | 0.11 |
| time | -0.04045 | 0.07373 | -0.549 | 0.59 |
| Location*time | 0.07099 | 0.10427 | 0.681 | 0.50 |
| Bacteria (Simpson Diversity) | | | | |
| Intercept | 9.87E-01 | 2.39E-04 | 4127.785 | **< 2e-16** |
| Location (SK) | -1.26E-02 | 3.38E-04 | -37.107 | **6.00E-10** |
| time | 2.46E-06 | 5.88E-06 | 0.418 | 0.68 |
| Location*time | -1.37E-05 | 8.32E-06 | -1.646 | 0.10 |
| Bacteria (Evenness) | | | | |
| Intercept | 8.48E-01 | 1.16E-03 | 731.63 | **< 2e-16** |
| Location (SK) | -5.97E-02 | 1.64E-03 | -36.444 | **5.00E-13** |
| time | 1.17E-06 | 3.44E-05 | 0.034 | 0.97 |
| Location*time | -5.78E-05 | 4.87E-05 | -1.188 | 0.24 |
| *Bacteria (Planctomycetes)* | | | | |
| Bacteria (OTU Number) | | | | |
| Intercept | 357.46294 | 3.70663 | 96.439 | **< 2e-16** |
| Location (SK) |  | 5.26272 |  |  |
|  | 0.96834 |  | 0.184 | 0.86 |
| time | -0.09654 | 0.10809 | -0.893 | 0.37 |
| Location*time | 0.05685 | 0.15294 | 0.372 | 0.71 |
| Bacteria (Simpson Diversity) | | | | |
| Intercept | 9.81E-01 | 4.88E-04 | 2011.717 | **< 2e-16** |
| Location (SK) | 6.76E-03 | 6.93E-04 | 9.759 | **2.00E-06** |
| time | -9.68E-06 | 1.38E-05 | -0.702 | 0.49 |
| Location*time | 3.31E-06 | 1.95E-05 | 0.17 | 0.87 |
| Bacteria (Evenness) | | | | |
| Intercept | 8.65E-01 | 1.78E-03 | 486.944 | **< 2e-16** |
| Location (SK) | 1.61E-02 | 2.52E-03 | 6.393 | **8.00E-09** |
| time | -8.35E-06 | 5.77E-05 | -0.145 | 0.89 |
| Location*time | 2.73E-05 | 8.17E-05 | 0.334 | 0.74 |
| *Bacteria (Proteobacteria)* | | | | |
| Bacteria (OTU Number) | | | | |
| Intercept | 254.12077 | 2.64762 | 95.981 | **< 2e-16** |
| Location (SK) | 15.28186 | 3.74431 | 4.081 | **1.00E-06** |
| time | -0.01691 | 0.08605 | -0.197 | 0.85 |
| Location*time | 0.02399 | 0.12169 | 0.197 | 0.84 |
| Bacteria (Simpson Diversity) | | | | |
| Intercept | 8.70E-01 | 2.34E-03 | 372.26 | **< 2e-16** |
| Location (SK) | 9.37E-02 | 3.30E-03 | 28.351 | **3.00E-13** |
| time | 1.12E-04 | 7.34E-05 | 1.521 | 0.13 |
| Location*time | -1.53E-04 | 1.04E-04 | -1.47 | 0.15 |
| Bacteria (Evenness) | | | | |
| Intercept | 6.72E-01 | 2.71E-03 | 248 | **< 2e-16** |
| Location (SK) | 1.17E-01 | 3.83E-03 | 30.551 | **< 2e-16** |
| time | 1.50E-04 | 8.81E-05 | 1.7 | 0.09 |
| Location*time | -2.05E-04 | 1.25E-04 | -1.645 | 0.10 |
|  | | | | |
| *Fungi (Ascomycota)* | | | | |
| Fungi (OTU Number) | | | | |
| Intercept | 106.49359 | 9.55558 | 11.145 | **<2e-16** |
| Location (SK) | 23.28686 | 13.51363 | 1.723 | 0.09 |
| time | -0.2996 | 0.31055 | -0.965 | 0.34 |
| Location*time | 0.06697 | 0.43918 | 0.152 | 0.88 |
| Fungi (Simpson Diversity) | | | | |
| Intercept | 0.9483872 | 0.0100942 | 93.953 | **7.00E-13** |
| Location (SK) | -0.0387191 | 0.0142754 | -2.712 | **0.03** |
| time | -0.0004032 | 0.000245 | -1.646 | 0.10 |
| Location*time | 0.0001199 | 0.0003465 | 0.346 | 0.73 |
| Fungi (Evenness) | | | | |
| Intercept | 0.8026188 | 0.011429 | 70.227 | **1.00E-16** |
| Location (SK) | -0.0937333 |  | -5.799 | **2.00E-04** |
|  |  | 0.016163 |  |  |
| time | -0.0003642 | 0.0003224 | -1.13 | 0.26 |
| Location*time | 0.0002313 | 0.000456 | 0.507 | 0.61 |
| *Fungi (Basidiomycota)* | | | | |
| Fungi (OTU Number) | | | | |
| Intercept | 20.277528 | 1.987682 | 10.202 | **<2e-16** |
| Location (SK) | 4.303015 | 2.811006 | 1.531 | 0.13 |
| time | 0.001555 | 0.064598 | 0.024 | 0.98 |
| Location*time | -0.008884 | 0.091355 | -0.097 | 0.92 |
| Fungi (Simpson Diversity) | | | | |
| Intercept | 0.6746623 | 0.0239429 | 28.178 | **< 2e-16** |
| Location (SK) | 0.2015002 | 0.0338603 | 5.951 | **6.00E-08** |
| time | 0.0002316 | 0.0007781 | 0.298 | 0.77 |
| Location*time | -0.0004707 | 0.0011004 | -0.428 | 0.67 |
| Fungi (Evenness) | | | | |
| Intercept | 0.5782517 | 0.0227841 | 25.38 | **7.00E-11** |
| Location (SK) | 0.2097352 | 0.0322215 | 6.509 | **5.00E-05** |
| time | 0.0006263 | 0.0006613 | 0.947 | 0.35 |
| Location*time | -0.0011156 | 0.0009352 | -1.193 | 0.24 |
| *Fungi (Zygomycota)* | | | | |
| Fungi (OTU Number) | | | | |
| Intercept | 7.57143 | 1.39524 | 5.427 | **2.00E-06** |
| Location (SK) | 1.33528 | 1.44377 | 0.925 | 0.36 |
| time | 0.07143 | 0.15798 | 0.452 | 0.65 |
| Location*time | -0.0774 | 0.15844 | -0.489 | 0.63 |
| Fungi (Simpson Diversity) | | | | |
| Intercept | 0.565336 | 0.040193 | 14.066 | **2.00E-15** |
| Location (SK) | 0.098609 | 0.041915 | 2.353 | **0.03** |
| time | 0.004326 | 0.004403 | 0.983 | 0.33 |
| Location*time | -0.004009 | 0.004416 | -0.908 | 0.37 |
| Fungi (Evenness) | | | | |
| Intercept | 0.5217674 | 0.0576722 | 9.047 | **5.00E-07** |
| Location (SK) | 0.0809379 | 0.0617587 | 1.311 | 0.22 |
| time | 0.0015105 | 0.005505 | 0.274 | 0.79 |
| Location*time | -0.0004732 | 0.0055211 | -0.086 | 0.93 |

**Table S5.** Analyses, including PERMANOVA, GLM of diversity metrics, and GLM (with liquid nitrogen as the storage reference), of qPCR measurements, examining impact of batch order in one extraction within Kansas samples.

| **a. PERMANOVA** | | | | | |
| --- | --- | --- | --- | --- | --- |
|  |  | Parameter |  | R^2^ | *p* value |
| Bacteria | | | | | |
|  |  | storage |  | 0.13554 | 0.17 |
|  |  | order |  | 0.04379 | 0.21 |
| Fungi | | | | | |
|  |  | storage |  | 0.08572 | 0.32 |
|  |  | order |  | 0.07968 | **1.20E-03** |
|  | | | | | |
| **b. GLM Diversity Metrics** | | | | | |
| Bacteria (OTU Number) | | | | | |
| Parameter | Estimate (coefficient) | | Std. Error | t-value | *p* value |
| Intercept | 1.97E+03 | | 2.24E+02 | 8.794 | **6.29E-10** |
| cooler | -86.62 | | 1.74E+02 | -0.497 | 0.62 |
| RNAlater | -297.01 | | 2.92E+02 | -1.017 | 0.32 |
| room temperature | 148.62 | | 1.74E+02 | 0.853 | 0.40 |
| order | 11.9 | | 1.50E+01 | 0.791 | 0.44 |
| Bacteria (Simpson Diversity) | | | | | |
| Intercept | 0.897743 | | 3.34E-02 | 26.911 | **1.27E-09** |
| cooler | -3.07E-02 | | 2.59E-02 | -1.184 | 0.27 |
| RNAlater | 7.49E-03 | | 4.34E-02 | 0.172 | 0.87 |
| room temperature | -1.76E-02 | | 2.59E-02 | -0.678 | 0.52 |
| order | 0.001052 | | 0.002235 | 0.47 | 0.65 |
| Bacteria (Evenness) | | | | | |
| Intercept | 0.634387 | | 0.046886 | 13.53 | **1.50E-14** |
| cooler | -0.047086 | | 0.036417 | -1.293 | 0.21 |
| RNAlater | 0.010665 | | 0.061035 | 0.175 | 0.86 |
| room temperature | -0.016455 | | 0.036417 | -0.452 | 0.66 |
| order | 0.002025 | | 0.003142 | 0.645 | 0.52 |
|  | | | | | |
| Fungi (OTU Number) | | | | | |
| Intercept | 212.381 | | 100.99 | 2.103 | **0.04** |
| cooler | 7.332 | | 78.441 | 0.093 | 0.93 |
| RNAlater | -24.558 | | 131.466 | -0.187 | 0.85 |
| room temperature | 167.779 | | 78.441 | 2.139 | **0.04** |
| order | 6.012 | | 6.768 | 0.888 | 0.38 |
| Fungi (Simpson Diversity) | | | | | |
| Intercept | 0.91694 | | 0.060024 | 15.276 | **5.63E-16** |
| cooler | -0.057637 | | 0.046622 | -1.236 | 0.23 |
| RNAlater | -0.073633 | | 0.078138 | -0.942 | 0.35 |
| room temperature | 0.008482 | | 0.046622 | 0.182 | 0.86 |
| order | 0.002897 | | 0.004023 | 0.72 | 0.48 |
| Fungi (Evenness) | | | | | |
| Intercept | 0.571266 | | 0.146288 | 3.905 | **4.75E-04** |
| cooler | -0.076438 | | 0.071927 | -1.063 | 0.30 |
| RNAlater | 0.103168 | | 0.120549 | 0.856 | 0.40 |
| room temperature | 0.060355 | | 0.071927 | 0.839 | 0.41 |
| order | 0.00466 | | 0.006206 | 0.751 | 0.46 |
|  | | | | | |
| **c. GLM of qPCR measurements** | | | | | |
| Parameter | Estimate (coefficient) | | Std. Error | t-value | *p* value |
| Bacteria (16S) | | | | | |
| Intercept | 1.06E+08 | | 4.49E+08 | 0.235 | 0.81 |
| cooler | -5.40E+08 | | 5.20E+08 | -1.038 | 0.30 |
| RNAlater | -8.36E+08 | | 5.29E+08 | -1.581 | 0.11 |
| room temperature | 1.09E+07 | | 5.44E+08 | 0.02 | 0.98 |
| order | 1.01E+08 | | 1.86E+07 | 5.423 | **5.87E-08** |
|  | | | | | |
| Fungi (ITS) | | | | | |
| Intercept | -2.15E+06 | | 2.99E+06 | -0.72 | 0.48 |
| cooler | -1.07E+06 | | 3.60E+06 | -0.297 | 0.77 |
| RNAlater | 1.77E+06 | | 3.65E+06 | 0.485 | 0.64 |
| room temperature | 5.02E+06 | | 3.72E+06 | 1.348 | 0.21 |
| order | 4.70E+05 | | 1.13E+05 | 4.175 | **2.99E-05** |

**Figure S1. Rarefaction curves for bacterial and fungal OTUs for (A) bacteria storage (6228 OTUs, samples rarified to 27758), (B) fungi storage (2103 OTUs, samples rarified to 36304), (C) bacteria cooler DNA extract thaw time (6474 OTUS, samples rarified to 20655) and (D) fungi cooler DNA extract thaw time (2157 OTUS, samples rarified to 37628). Curves show saturation of sequences, indicating sufficient sequencing.**

**Supplementary Methods**

**Amplicon library preparation and Illumina Miseq Sequencing**

*1. Bacteria (16S)*

Amplicon library preparation was completed at the Genome Quebec Innovation Center using a two-step PCR procedure for the 16S rRNA gene. The first step involved amplifying bacterial DNA using the tagged primers 515-F and 806-R, and second step added barcodes and Illumina adapters. The initial 25 µl PCR included 0.5 U FastStart High Fidelity (Roche), 10X PCR buffer with 18 mM MgCl_2_ (Roche), 5% DMSO (Roche), 0.2 mM dNTP mix (FroggaBio), 0.6 µM of each primer (515F-CS1 and 806R-CS2), and 1 µl of DNA diluted 1/10. The PCR conditions consisted of 94°C for 2 min, 33 cycles at 94°C for 30 s, 58°C for 30 s, and 72°C for 30 s, followed by 72°C for 7 min. Verification of amplification was assessed on 2% agarose gel. The PCR product was diluted 1/100 for the second PCR step to add 10-bp barcodes and Illumina adapter sequences in 18 µl reactions. The PCR conditions for the second step consisted of 95°C for 10 min, 15 cycles at 95°C for 15 s, 60°C for 30 s, and 72°C for 60 s, followed by 72°C for 3 min. Verification of successful barcode incorporation was assessed on 2% agarose gel. Amplicons were quantified with Quant-iT™ PicoGreen® dsDNA Assay Kit (Life Technologies) and libraries generated by pooling the same quantity (ng) of each sample. Libraries were purified with a ratio of 0.85 sparQ Pure Mag Beads (Quantabio). Libraries were then quantified using Kapa SYBR Fast Universal Kit (Kapa Biosystems) and average fragment size was determined using a LabChip GX instrument (PerkinElmer). Prior to sequencing, 10% of Phix control library was spiked into the amplicon library at a final concentration of 8 pM to improve the unbalanced base composition. Paired-end sequencing was performed with a MiSeq reagent kit v2 (500- cycle) (Illumina).

*2. Fungi (ITS1)*

Amplicon library preparation was completed at the Genome Quebec Innovation Center using a two-step PCR procedure for the fungal ITS1 region. The first step involved amplifying fungal DNA using the tagged primers ITS1F and 58A2R, and second step added barcodes and Illumina adapters. The initial 8 µl PCR included 0.16 U HotStarTaq (Qiagen), 10X PCR buffer with 15 mM MgCl_2_ (Qiagen), 5% DMSO (Roche), 0.2 mM dNTP mix (FroggaBio), 0.6 µM of each primer (ITS1F-CS1 and 58A2R-CS2), and 1 µl of DNA diluted 1/100. The PCR conditions consisted of 96°C for 15 min, 35 cycles at 96°C for 30 s, 52°C for 30 s, and 72°C for 60 s, followed by 72°C for 10 min. Verification of amplification was assessed on 2% agarose gel. The PCR product was diluted 1/100 for the second PCR step to add 10-bp barcodes and Illumina adapter sequences in 18 µl reactions. The PCR conditions for the second step consisted of 95°C for 10 min, 15 cycles at 95°C for 15 s, 60°C for 30 s, and 72°C for 60 s, followed by 72°C for 3 min. Verification of successful barcode incorporation was assessed on 2% agarose gel. Amplicons were quantified with Quant-iT™ PicoGreen® dsDNA Assay Kit (Life Technologies) and libraries generated by pooling the same quantity (ng) of each sample. Libraries were purified with a ratio of 0.85 sparQ Pure Mag Beads (Quantabio). Libraries were then quantified using Kapa SYBR Fast Universal Kit (Kapa Biosystems) and average fragment size was determined using a LabChip GX instrument (PerkinElmer). Prior to sequencing, 10% of Phix control library was spiked into the amplicon library at a final concentration of 10 pM to improve the unbalanced base composition. Paired-end sequencing was performed with a MiSeq reagent kit v3 (600- cycle) (Illumina).
